# The Effectiveness of Treatments for Cocaine Dependence in Schizophrenic Patients: A Systematic Review

**DOI:** 10.2174/1570159X11311050003

**Published:** 2013-09

**Authors:** Pamela Sabioni, Anna Carolina Ramos, Jose Carlos F. Galduróz

**Affiliations:** Department of Psychobiology (Departamento de Psicobiologia), Universidade Federal de São Paulo, Brazil

**Keywords:** Cocaine, Dependence, Treatment, Schizophrenia.

## Abstract

**Objectives:**

To evaluate and compare the effectiveness of available treatments for cocaine dependence in schizophrenic patients.

**Method:**

We searched articles published between May 2002 and June 2012 in the following databases: Scopus, Pubmed and Web of Knowledge. The key words utilised were “schizophrenia”, "dementia praecox", "schizophrenic disorder", "cocaine related disorder", "cocaine abuse", "cocaine addiction", "cocaine dependence", “treatment”, “therapeutic”, and "drug therapy”.

**Selection of studies and data extraction:**

Original articles in English, Portuguese and Spanish were selected. Controlled, double-blind and open-label studies involving only human subjects were included in this review.

**Data Synthesis:**

We found studies on typical and atypical antipsychotics and one monoamine transporter antagonist. There were few indications of the effectiveness of atypical antipsychotic medications for the treatment of cocaine dependence in patients with schizophrenia.

**Conclusions:**

We suggest that further studies be conducted with atypical antipsychotic medicationsand greater methodological strictness, including using a placebo group in the studies, so that health professionals can determine the real effectiveness of this class of medication for the treatment of cocaine dependence in schizophrenic patients.

## INTRODUCTION

Schizophrenia is a severe mental disorder that affects approximately 24 million people worldwide, mainly those between 15 and 35 years of age [1]. The primary neurochemical model that explains the symptoms of schizophrenia is the dopaminergic theory, which asserts that a hyperactive dopaminergic system in the mesolimbic pathway is responsible for the positive symptoms of the disorder. The main symptoms in this phase are delusions, hallucinations and psychomotor agitation. The dopaminergic hypofunction in the mesocortical pathway, on the other hand, is related to the negative symptoms, which is reduced emotional, motivational and cognitive capacity [2, 3].

Substance abuse among individuals with schizophrenia is very high. Approximately 50% of the patients present lifetime abuse of some substance [4, 5]. The model of dysfunction in the circuit of the brain reward system is one theory that attempts to explain the high rate of co-morbidity of substance abuse and schizophrenia.

This theory suggests that the abuse of potentially addictive substances by individuals with schizophrenia is related to the individual’s difficulty in experiencing the normal levels of reward obtained from the environment and the drug’s capacity to reduce this deficit in the reward circuit [6, 7]. 

Other important theories postulate that schizophrenic subjects use drugs to self-medicate, to alleviate painful emotional states, to relieve the distress associated with mood disorders (such as depression and hypomania), and to counteract extrapyramidal effects [8-10]. Further evidence shows that chronic antipsychotic treatment might induce supersensitivity in the dopamine-mediated reward function. This supersensitivity can increase the incentive motivational properties of reward cues and contribute to the severity of drug addiction [11], which in turnworsens the course of the mental disorder, reduces adherence to treatment, increases the relapse rate, and can significantly impair the relationship between the patient and health professionals [12-15]. 

The high prevalence of cocaine abuse among schizophrenic patients represents a potential problem if we consider the debilitating effects of the drug on both acute symptoms and the course of the disorder. Cocaine abuse among this population generally has devastating consequences for the individual and a negative impact on society. Individuals with schizophrenia and cocaine abuse present an exacerbation of the positive and extra-pyramidal symptoms, and a decrease of negative symptoms and cognitive impairment, which frequently results in utilisation of psychiatric emergency services [16-18].

Some research attempts to find an effective cocaine treatment targeted at schizophrenic patients. Typical antipsychotics, which promote a potent blockage of D2 receptors, show little effectiveness in the treatment of dependence. In contrast, atypical antipsychotics, which act on serotoninergic receptors, dissociate from the D2 receptor faster than the typical antipsychotics, produce dopaminergic stimulation, increase the dopamine release in some brain areas, potentially improve both the negative and the positive symptoms of the disorder, and effectively cause no extra-pyramidal effects [19, 20].

Although the scientific literature includes some studies on the pharmacological treatment for cocaine dependence in schizophrenic patients, there is no well-established pharmacotherapy for this issue. Therefore, this systematic review aims to address new information on the effectiveness of current treatments for this clinical condition.

## METHODS

To identify the relevant articles for this review, we searched the databases Pubmed, Scopus and Web of Knowledge. The search words utilised were “schizophrenia”, "dementia praecox", "schizophrenic disorder", "cocaine related disorder", "cocaine abuse", "cocaine addiction", "cocaine dependence", “treatment”, “therapeutic”, and “drug therapy”. The articles were published between May 2002 and June 2012. All the original articles that reported pharmacological treatment for cocaine abuse or dependence in schizophrenic patients were included in this review. Only original articles in English, Portuguese and Spanish were selected. Controlled, double-blind and open-label studies involving only human subjects were included in this review. Two researchers conducted each phase to guarantee that all inclusion criteria were met. Fig. (**1**) outlines the total number of publications retrieved, the inclusion/exclusion criteria for the publications under review and the number of studies included in the final selection.

## RESULTS

We found studies that utilised only three groups of medication to treat cocaine dependence in patients: typical antipsychotics, atypical antipsychotics and a monoamine transporter antagonist (Table **1**). 

Sayers and colleagues (2005) [21] performed a double-blind study that compared the effects of haloperidol (a typical antipsychotic) to those of olanzapine (an atypical antipsychotic) in cocaine-abusing schizophrenic patients. The study lasted 26 weeks and involved 24 patients. The current antipsychotic medications were first halved and then gradually reduced over the first 1 to 2 weeks of the study after randomisation. Twelve patients took 10 mg to 20 mg of haloperidol/day, depending on their clinical evolution. The researchers performed an intention-to-treat analysis and compared differences in symptoms across the 26 weeks of the study for each subject. The proportion of positive results in the urine test for cocaine was significantly higher than the basal values for those patients. These values were obtained before the beginning of treatment, and the proportion as a binomial variable wascalculated based on the presence or absence of positive results during the study. The results showedan increase in the consumption of cocaine during the haloperidol treatment period. Theauthors performed an additional analysis of patients’ self-reports of craving, which revealed a significant decrease in relation to basal levels after six months for patients in the haloperidol group; this finding is an inverse association of these two measures. The other 12 patients received olanzapine (10 mg to 20 mg/day) and showed decreased cocaine use at the end of the study compared to basal levels, as assessed by urine screening test for cocaine. Conversely, self-reports on craving showed a significant increase in relation to the basal levels during the last four months of the study. Survival analysis based on the assignment groups was performed with the Kaplan-Meier estimator. The drop-out rate was similar in both groups, with seven patients remaining in each group at the end of the study. 

A second double-blind study of patients with schizophrenia and cocaine dependence was 6 weeks in length [22]; this study compared haloperidol (10 mg to 20 mg/day) with 15 patients and olanzapine (10 mg to 20 mg/day) with 16 patients. The Voris Cocaine Craving Questionnaire (VCCQ) and Positive and Negative Syndrome Scale (PANSS) scores of patients that completed the study showed improvement in the energy subscale of the VCCQ, whichevaluated emotionality after the haloperidol treatment and cocaine use intention elicited by an artificial cue. This result indicates that the subjects showed less cue-elicited craving after a videotape of people smoking cocaine and administering intravenous cocaine. Additionally, the subscale of intensity decreased. However, the author did not make this comparison. The patients who received olanzapine had significantly less craving in the subscale of energy; this finding demonstrates a decrease in emotionality and cocaine use intention when compared with the group that received haloperidol. 

Akerele and Levin (2007) [23] compared the effects of olanzapine to risperidone in 28 cocaine-abusing schizophrenic patients. Fourteen patients (11 of which were cocaine dependent) started the treatment with olanzapine at a dose of 5 to 20 mg/day, and six of them remained until the end of the 10-week study. The patients participated in a two-week cross-taper phase that tapered them off their previously prescribed medication. Time *versus* treatment interactions were evaluated for all outcome measures. Cocaine use was lower during the evaluation period, but no differences across groups were detected in either positive testing for cocaine use or craving scores. The other 14 patients (9 cocaine dependent) received risperidone (3 to 9 mg/day). Time-to-dropout between groups was compared using Kaplan-Meier survival curves and the long-rank test. Ten patients in this group remained until the end of the study. There were no differences in the positive tests for cocaine in urine or in the scale of craving between the two groups during the evaluation period. 

Risperidone was also utilised in an open study [24]. A total of 18 patients on typical neuroleptic treatment were recruited, and the patients assigned to the risperidone group were cross-tapered with that medication. Eight patients started the treatment with that medication (a maximum dose of 6 mg/day), while 10 patients were assigned to the treatment group with typical antipsychotics (chlorpromazine, fluphenazine and haloperidol). Nine patients completed the treatment (six with risperidone, three with typical antipsychotics) for up to 6 weeks.The study utilised a “last-observation-carried-forward” analysis and Student *t* test to measure group differences in craving.The patients who used risperidone presented a lower intensity of cue-elicited craving and less depression than those who took chlorpromazine. Additionally, 12.5% of the patients that used risperidone relapsed, compared with 70% that received typical antipsychotics (Fisher Exact test).

Aripiprazole was utilised in two studies. In an open study, the subjects were required to discontinue their current antipsychotic medication and begin oral aripiprazole. Ten cocaine dependent individuals with schizophrenia started an eight-week treatment with aripiprazole (10 mg to 15 mg/day). Six of them completed the treatment and showed a significant decrease in both cocaine use and craving [25]. This study analysed craving changes reported over time in 2 ways: 1) observed reduction in the Brief Substance Craving Scale (BSCS) scores for cocaine and alcohol over the study period and 2) comparison of mean baseline and week 8 craving scores for cocaine and alcohol, using Student *t* test for paired data and a 1-tailed test. Changes in the frequencies of cocaine positive urine samples due to the low frequency of positive urine during the treatment were evaluated with Fisher Exact test. Additionally, Pearson *r*assessed the associations between the BSCS and Brief Psychiatric Rating Scale (BPRS) measures.

In the second open study, McRae-Clark *et al*. (2009) [14] investigated aripiprazole as a treatment for patients that met the criteria for schizophrenia and schizoaffective disorder and presented alcohol, cocaine or marijuana abuse or dependence. Other antipsychotic or mood-stabilising medications were not allowed during the study. Among 13 patients with cocaine abuse or dependence, 11 had schizophrenia or schizoaffective disorder. Only six schizophrenic patients continued until the end of the study. Aripiprazole was administered at the dose of 15 mg to 30 mg/day for a period of eight weeks. The Wilcoxon signed-rank test examined the percentage of using days and the amount used, and the measured impact of the therapy on the psychiatric outcomes linear growth models were estimated. The Timeline Followback measured a significant reduction in cocaine use among the individuals with abuse or dependence, independent of the mental disorder diagnosed. 

A double-blind study evaluated the effect of mazindol, which is a potent blocker of monoamine transporters [26]. Twenty-four patients were randomly assigned to two groups (n=12 in each) and received either mazindol (1 mg/tid to 2 mg/tid) or placebo for a period of up to six weeks. All patients received antipsychotic medications.Only three patients in the mazindol group remained until the end of the study. This group showed no significant reduction in either craving or amount of cocaine used during the study.

## DISCUSSION 

The studies included in this review showed that typical antipsychotics and the monoamine transporter antagonist did not improve the symptoms of cocaine dependence in schizophrenic patients and sometimes even exacerbated them. However, studies showed that atypical antipsychotics, especially aripiprazole, effectively reduced cocaine use. In some cases, however, the same medication presented opposite results in relation to cocaine abuse or dependence. Olanzapine showed increased craving in one study, reduced craving in a second one and no effects in a third one [21-23]. Risperidone reduced craving and the relapse rate in one study, but not in another [18, 23]. 

The only medication utilised in two different studies with similar results wasAripiprazole; decreased craving and consumption were reported in one study, and the other study reported less money was spent on cocaine purchases [14, 25]. 

The studies show great methodological heterogeneity. First, considering their classification, four studies are double-blind, while three studies are open-label. This is an important factor in treatment outcomes because patient and doctor knowledge of treatment type might influence the response to medication. 

Another limitation of the general interpretation of data was the lack of placebo-controlled groups. Among the studies included in this review, only the study by Perry Jr. *et al*. (2005) [27] used a placebo. The utilisation of placebo-controlled groups has been largely discussed in the medical literature; because there seems to be no established consensus on this issue, some of the main points are stressed below.

Fleischhaker *et al*. (2003) [28] stated two main considerations in the discussion of placebo use in clinical trials for schizophrenia. One issue is the unethical practice of giving patients a placebo when effective treatments for the disorder exist. Another concern is that determination of the efficacy of new drugs is more reliably accomplished in a placebo-controlled design. Studies such as Woods *et al*. (2005) aimed to compare placebo with active control design and found that both protocols have biases. While placebo-controlled trials are biased towards smaller improvements, active-controlled ones are biased towards larger improvements [29].

Another alternative evaluated is the use of the experimental drugs and placebo in combination with another antipsychotic. This approach would solve both the placebo group problem and the ethical question of not treating patients [30]. This type of protocol was utilised by Perry Jr. *et al*., (2005) [26]; previous medications were continued in this study, and the patients were randomly allocated to take either placebo or mazindol. A problem with this study was the lack of information about patient simultaneous medication use.

Second, the treatment duration and the number of patients involved differed. Only one study extended beyond 10 weeks (26 weeks). Such short durations may have prevented detection of potentially effective longer-term treatments. This situation might be a negative factor for results because a short treatment may not be enough to promote changes in patient cocaine dependence or abuse. The sample size in all the studies was very small, and three to 10 patients per group remained until the end of the treatment. Both cocaine dependent and schizophrenic patients have generally high drop-out rates [15, 31]. The rates of drop-out in the studies analysed represented a potential complication of data interpretation. Such small samples could compromise the applicability of the therapy to other populations.

A third problem in the data interpretation was the lack of clarity of the information presented in the studies. McRae-Clark and colleagues (2009) [14], for example, do not indicate whether the 13 cocaine abusing or dependent patients presented the same pattern as other drugs. That study did not report how many of those patients finished the treatment. In another study[14], the criterion of improvement was the amount money spent on the purchase of the drug, which is a measure that cannot be very reliable.

Many patients with schizophrenia frequently switch their antipsychotic medications and commonly take them concomitantly with other psychotropic medications. Additionally, patients are exposed to both typical and atypical antipsychotics throughout their lifetime, which complicates the study of efficacy of one antipsychotic treatment versus another. The studies selected do not mention medical assessments.

The countless limitations presented in the investigation of this issue highlight the difficulty in the treatment of those two concomitant pathologies. The scant effective medications that handle psychotic symptoms and treat dependence and the low adherence to available therapies make research on this issue challenging.

The intersection between the neurobiology underlying schizophrenia and drug dependence might help us understand this phenomenon. Because both pathologies involve disorders in the dopaminergic system, the key to their treatment may lie either in the overlap or in the peculiarities of each single disorder.

Cocaine is a frequent drug of choice for schizophrenics, possibly due to its high dopaminergic stimulation [32-34]. The potent stimulant effects result from the blockage of dopaminergic transporters, which is a process that entails a large, rapid increase in the levels of dopamine available in the mesolimbic system [35, 36]. This dopaminergic action is associated with euphoriant effects, paranoia and increased psychomotor activity related to drug consumption [37, 38]. A possible hypothesis is that cocaine use might re-establish the dopaminergic levels that are often diminished by the long term use of the antipsychotic medication.

Typical antipsychotics are potent blockers of D2 receptors of long duration, primarily in the mesolimbic pathway [3]. This action produces both therapeutic effects that reduce the positive symptoms of the disease and side effects triggered by the blockage of those receptors in the other dopaminergic pathways, including extrapyramidal symptoms, tardive dyskinesia and prolactin elevation. The blockage of those receptors in the mesolimbic pathway is additionally responsible for the inhibition of the reward system, primarily the nucleus accumbens, which can cause apathy, anhedonia, lack of motivation and loss of interest and pleasure in schizophrenic patients. The blockage of D2 receptors in the mesocortical pathway may cause or aggravate negative disease symptoms, and dopamine levels may already be deficient [39, 40]. 

Atypical antipsychotics do not cause as many extrapyramidal effects and improve the negative symptoms of the disease [41, 42]. These drugs have a more complex mechanism of action, including effects on the serotoninergic and dopaminergic systems. 

This medication might act in the serotoninergic pathway with two different mechanisms that yield the same therapeutic effects. The antagonism of the serotoninergic receptors 5HT2A or the partial agonism of the 5HT1A receptors can produce dopaminergic stimulation that increases the dopamine release in some brain areas [41, 43, 44]. This process accounts for a low incidence of extrapyramidal symptoms and an improvement of the negative symptoms. Moreover, the receptors can improve the positive symptoms through the reduction of glutamate release in the mesocorticolimbic system. The increase of glutamate in this pathway is usually responsible for hallucinations and other positive symptoms of the disorder due to the hyperstimulation of the dopaminergic system in the mesolimbic pathway [45].

Additionally, atypical antipsychotics dissociate from the D2 receptor faster than typical ones because the latter have a long binding duration. Theoretically, these drugs bind to the receptor long enough to exert a therapeutic action but not long enough to produce the side effects caused by typical antipsychotics [40]. 

Some atypical antipsychotics, such as aripiprazol [46], may also be classified as partial agonists of D2 receptors. The moderate improvement in the cocaine abuse and dependence of schizophrenic patients observed in some studies may be due to the regulation mechanism that atypical antipsychotics promote in the mesolimbic dopaminergic system. Specifically, these atypical antipsychotics do not decrease the dopaminergic stimulation of the reward system to the point that the individual would need the drug as an alternative compensation mechanism.

Additionally, some preclinical studies showed evidence that an atypical antipsychotic may be useful in the treatment of cocaine dependence. Some medications of this class of drugs were capable of reducing the self-administration and reinforcement effects of cocaine in rats and monkeys [47-50].

We recommend further investigation of the effect of atypical antipsychotic medications for cocaine abusing/dependent patients with schizophrenia. The studies should be controlled, with larger sample sizes and a long duration to identify the effect of the drugs used.

## Figures and Tables

**Fig. (1):**
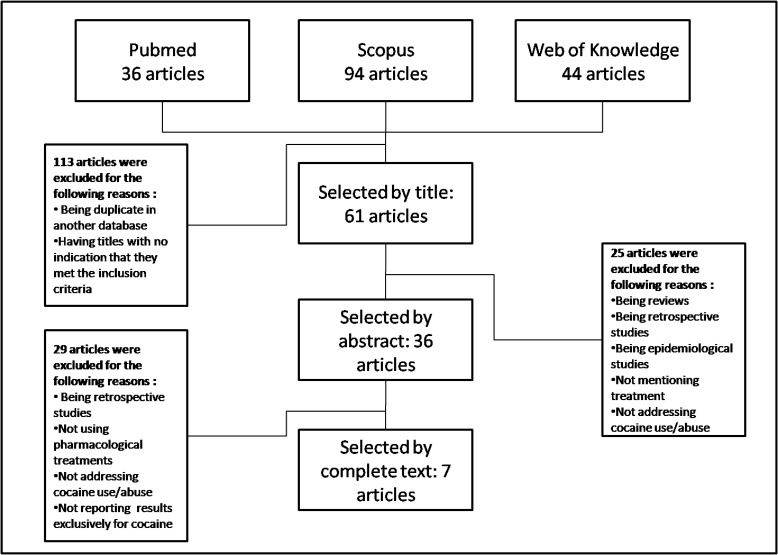
Flowchart of the method utilised for article selection.

**Table 1.** Summary of the Methodology and the Results of the Articles Selected

Study	Type	Treatment/dose	Duration	Number of Patients		Results
				Beginning	End	
Sayers *et al*., 2005 [21]	Double-blind	Haloperidol 10 mg/day to 20 mg/day Olanzapine 10 mg/day to 20 mg/day	26 weeks	12 12	7 7	Increase of cocaine consumption Decrease of craving Decrease of cocaine consumption Increase of craving
Smelson *et al*., 2006 [22]	Double-blind	Haloperidol 10 mg/day to 20 mg/day Olanzapine 10 mg/day to 20 mg/day	6 weeks	15 16	10 8	Increase of craving Decrease of craving
Akerele & Levin, 2007 [23]	Double-blind	Olanzapine 5 mg/day to 20 mg/day Risperidone 3 mg/day to 9 mg/day	10 weeks	14 14	6 10	Small decrease in cocaine consumption in the group treated with olanzapine compared to the group treated with risperidone. No decrease of craving in either treatment
Smelson *et al*., 2002 [24]	Open-label	Risperidone 6 mg/day Typical antipsychotics	6 weeks	8 10	6 3	Group treated with risperidone presented less craving and lower relapse rate than group treated with chlorpromazine
Beresford *et al*., 2005 [25]	Open-label	Aripiprazole 10 mg/day to 15 mg/day	8 weeks	10	6	Decrease of cocaine consumption and craving
McRae-Clark *et al*., 2009 [26]	Open-label	Aripiprazole 15 mg/day to 30 mg/day	8 weeks	13*	?	Decrease in the amount of money spent on the purchase of cocaine
Perry Jr. *et al*., 2005 [27]	Double-blind	Mazindol 1 mg/tid to 2 mg/tid Placebo	6 weeks	12 12	3 5	No decrease of craving No decrease of consumption

* This number represents the cocaine abusing or dependent patients included in the analysis rather than the schizophrenic patients only. The number of patients that completed the study treatment is not known.
